# Dynamic immune markers predict HIV acquisition and augment associations with sociobehavioral factors for HIV exposure

**DOI:** 10.1016/j.isci.2022.105632

**Published:** 2022-11-19

**Authors:** Rachel A. Bender Ignacio, Sayan Dasgupta, Rogelio Valdez, Urvashi Pandey, Siavash Pasalar, Ricardo Alfaro, Florian Hladik, Germán Gornalusse, Javier R. Lama, Ann Duerr

**Affiliations:** 1 Division of Allergy and Infectious Diseases, Department of Medicine, University of Washington, Seattle, WA 98104, USA; 2 Vaccine and Infectious Diseases Division, Fred Hutchinson Cancer Center, Seattle, WA 98109, USA; 3 Case Western Reserve University School of Medicine, Cleveland, OH 44106, USA; 4 Department of Obstetrics and Gynecology, University of Washington, Seattle, WA 98195, USA; 5 Centro de Investigaciones Tecnológicas Biomédicas y Medioambientales, Universidad Nacional Mayor de San Marcos, Bellavista, Lima 07006, Peru; 6 Asociación Civil Impacta Salud y Educación, Lima 15063, Peru; 7 Department of Global Health, University of Washington, Seattle, WA 98195, USA

**Keywords:** Health sciences, Virology, Machine learning

## Abstract

Prior studies attempting to link biomarkers of immune activation with risk of acquiring HIV have relied on cross sectional samples, most without proximity to HIV acquisition. We created a nested case-control study within the *Sabes* study in Peru, and assessed a panel of plasma immune biomarkers at enrollment and longitudinally, including within a month of diagnosis of primary HIV or matched timepoint in controls. We used machine learning to select biomarkers and sociobehavioral covariates predictive of HIV acquisition. Most biomarkers were indistinguishable between cases and controls one month before HIV diagnosis. However, levels differed between cases and controls at study entry, months to years earlier. Dynamic changes in IL-2, IL-7, IL-10, IP-10 and IL-12, rather than absolute levels, jointly predicted HIV risk when added to traditional risk factors, and there was modest effect modification of biomarkers on association between sociobehavioral risk factors and HIV acquisition.

## Highlights

• Biomarkers differ at baseline in those who acquired HIV versus controls
• Cytokine profiles not different in cases versus controls immediately before HIV acquisition
• Changes in cytokines over time most predictive of HIV acquisition
• Cytokines modify effect of sociobehavioral risk factors on HIV acquisition

## Graphical abstract

**Figure:**
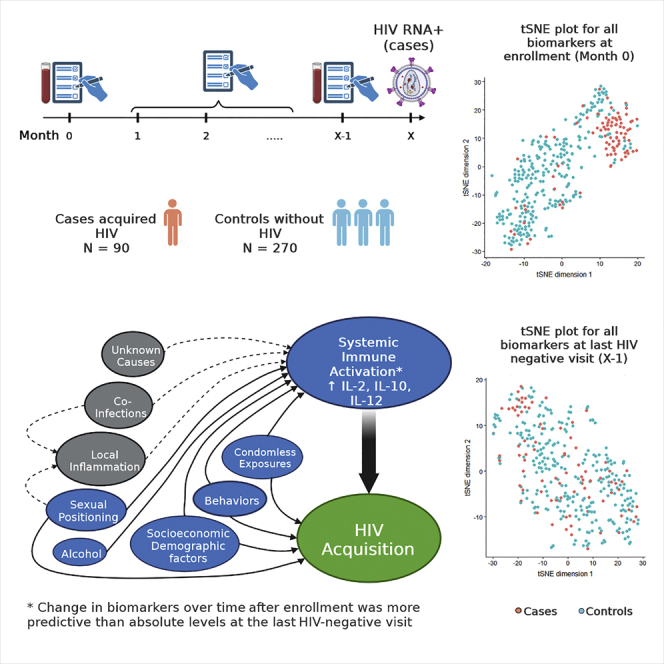

## Introduction

The possibility that certain systemic immune responses increase HIV susceptibility was first documented in the Step (HVTN 502/504) trial, in which some vaccinees who received the recombinant MRK adenovirus type 5 vector HIV-1 clade B gag (MRKAd5 gag HIV Type 1) vaccine had a transient 2-fold increased risk of HIV acquisition.^1^ Of importance, ELISpot mock responses (IFN-ɣ secretion in the absence of antigen-specific stimulation), but not HIV-antigen-stimulated responses, were directly correlated with risk of HIV acquisition among MRKAd5 gag HIV Type 1 vaccinees.^2^ CAPRISA-004 assessed efficacy of a vaginal tenofovir gel for HIV prevention and collected blood from participants at baseline, 3, 12 and 24 months after enrollment.^3^ A nested case-control study within this cohort identified immune activation in the last pre-infection sample, defined as natural killer (NK) cell CD38/HLA-DR positivity, elevated platelets, and elevated IL-2, IL-7, IL-12, and TNF-α, as risk factors for HIV acquisition, independent of HSV-2 serostatus and sexual exposure.^4^ However, the study was limited by its small size and infrequent sampling, with gaps of up to one year between samples; its results are therefore controversial.^4^ The Partners in Prevention study of tenofovir-based oral pre-exposure prophylaxis targeting HIV-discordant couples similarly evaluated cytokines in blood samples from seroconverting partners taken a mean of 3 months before HIV acquisition, compared to samples from partners who remained HIV-uninfected.^5^ Genital tract infections, HIV viral load in the transmitting partner, age, and unprotected sex were associated with HIV acquisition. IL-10 and IP-10 were the only cytokines associated with HIV acquisition; the predictive biomarkers from CAPRISA-004 were not confirmed.^6^ Most studies on immune correlates of risk have been conducted in serodiscordant heterosexual couples, most of which have focused on cisgender women whose exposure is via receptive vaginal intercourse, and genital inflammation.^4^^,^^7^

Peripheral blood markers reported to be associated with HIV acquisition in other studies are not surprisingly involved in T cell activation and maintenance, and in antiviral responses.^8^^,^^9^^,^^10^ Elevations in these markers have to date not been correlated with demographic, behavioral, or exposure data that could provide explanations for the risk associated with these immune profiles. Identifying high-risk immunologic profiles for HIV acquisition is important not only for developing vaccines that avoid these pitfalls, as occurred in the MRKAd5 gag HIV-1 vaccine, but also for identifying ways to modify exposures or pathways to reduce risk of HIV acquisition.

Longitudinal cryopreserved samples, including specimens taken shortly before HIV acquisition, are exceedingly rare. We took advantage of a unique cohort with frequent HIV testing and stored samples to study risk factors for acquisition. The *Sabes* study was a treatment-*as*-prevention (TasP) study in Lima, Peru, that followed a cohort of persons assigned male at birth and at elevated risk for HIVfor two years with frequent HIV testing by serology and RNA, to identify HIV infection shortly after acquisition. Those with incident HIV acquisition were randomized to begin antiretroviral therapy during primary vs post-primary infection^11^ (before WHO recommendations for immediate universal HIV treatment^12^). Stored specimens collected at multiple timepoints before infection in this study allowed serial evaluation of potential markers of HIV acquisition risk in a nested case-control (1:3) study of persons who did or did not acquire HIV.

## Results

Characteristics of cases who acquired HIV and matched controls who did not are presented in Table 1. Participants were young (median age for cases 25 years (IQR 20, 29) versus 28 years (23.0, 36.0 for controls). The majority identified as MSM, while 12.2% of cases and 17.4% of controls identified as transfeminine, and 0.7% of controls identified as non-binary/other identity. The median time from enrollment to HIV diagnosis in cases was 361 days (IQR 180, 517). The median interval between the last HIV negative visit (X-1) and HIV diagnosis (X) was 35 days (IQR 29, 44) with median interval between calculated estimated date of detectable infection (EDDI)^13^ and the last HIV negative visit of 5.5 days (IQR 0.25, 17.75). Among Sabes participants, moderate and high alcohol use was common (the median AUDIT score was 11 and 9 respectively, putting most participants in the “hazardous” consumption category); less than 10% of participants reported use of cocaine, marijuana, opiates, or other substance use among a subset who responded to an in-depth substance use questionairre.^14^

**Table 1:** Characteristics of Participants who did or did not subsequently acquire HIV

Characteristics at entry	Cases N = 90	Controls N = 270
**Age**		

median (IQR)	25 (20.3, 29.0)	28 (23.0, 36.0)

**Race/ethnicity**		

Latinx, mestizo (multiracial)	90 (100%)	270 (100%)

**Post-secondary education/training**		

Yes	66 (73.3%)	139 (51.5%)

**Income above minimum wage**		

Yes^a^	53 (58.9%)	134 (49.8%)

**Participate in sex work**		

Yes	20 (22.2%)	117 (43.3%)

**Gender identity**^b^		

Cisgender Male	79 (87.8%)	221 (81.9%)
Transgender female	11 (12.2%)	47 (17.4%)
Non-binary or other	0 (0.0%)	2 (0.7%)

**Sexual orientation**		

Heterosexual (non-queer identified MSM)	0 (0.0%)	15 (5.6%)
Bisexual	33 (36.7%)	77 (28.5%)
Homosexual	46 (51.1%)	129 (47.8%)
Other (inc. transwomen)	11 (12.2%)	47 (17.4%)

**AUDIT score**		

median (IQR)	11 (5, 17)	9 (6, 15)

**Report current CLAI**		

Yes	79 (87.8%)	209 (77.4%)

**CLAI acts in**the **30days before X visit**^c^		

Mean; median (IQR)	1.6; 0.0 (0.0, 2.0)	0.9; 0.0 (0.0, 0.0)

**Sexual Role**		

Insertive only	10 (11.1%)	84 (31.1%)
Receptive only	25 (27.8%)	81 (30.0%)
Versatile	55 (61.1%)	103 (38.1%)

**Days to HIV diagnosis (X)**		

median (IQR)	361 (179.8, 516.5)	359.5 (185.5, 498.8)

**Days between X-1 sample and X**		

median (IQR)	35 (29, 44)	33 (28, 38)

**Days between EDDI**^d^**and X**		

median (IQR)	29 (18, 35)	–

MSM, Men who have Sex with Men; AUDIT, Alcohol Use Disorders Identification Test; CLAI, condomless anal intercourse; X-1 is the sample selected from the month before X or the last HIV-negative sample, where X is the first HIV positive by HIV RNA and/or Ab point-of-care test or matched visit in controls

a During 2015, the Peruvian national minimum wage was 750 PEN (Peruvian Nuevo Soles), or about $235 US dollars/month.

b All participants were assigned male at birth by eligibility criteria of parent study.

c Participant-reported count of total number of CLAI acts with any non-main partner (insertive or receptive) in the 30 days before the visit. See STAR methods for rationale and details.

d EDDI, or Estimated Date of Detectable Infection, is calculated from a published calculator that uses all available HIV test results and methods to calculate the most probable date of HIV acquisition, including uncertainty estimates for First Possible and Last Possible Date of Infection.

### Biomarkers over time and HIV risk

**Figure 1:**
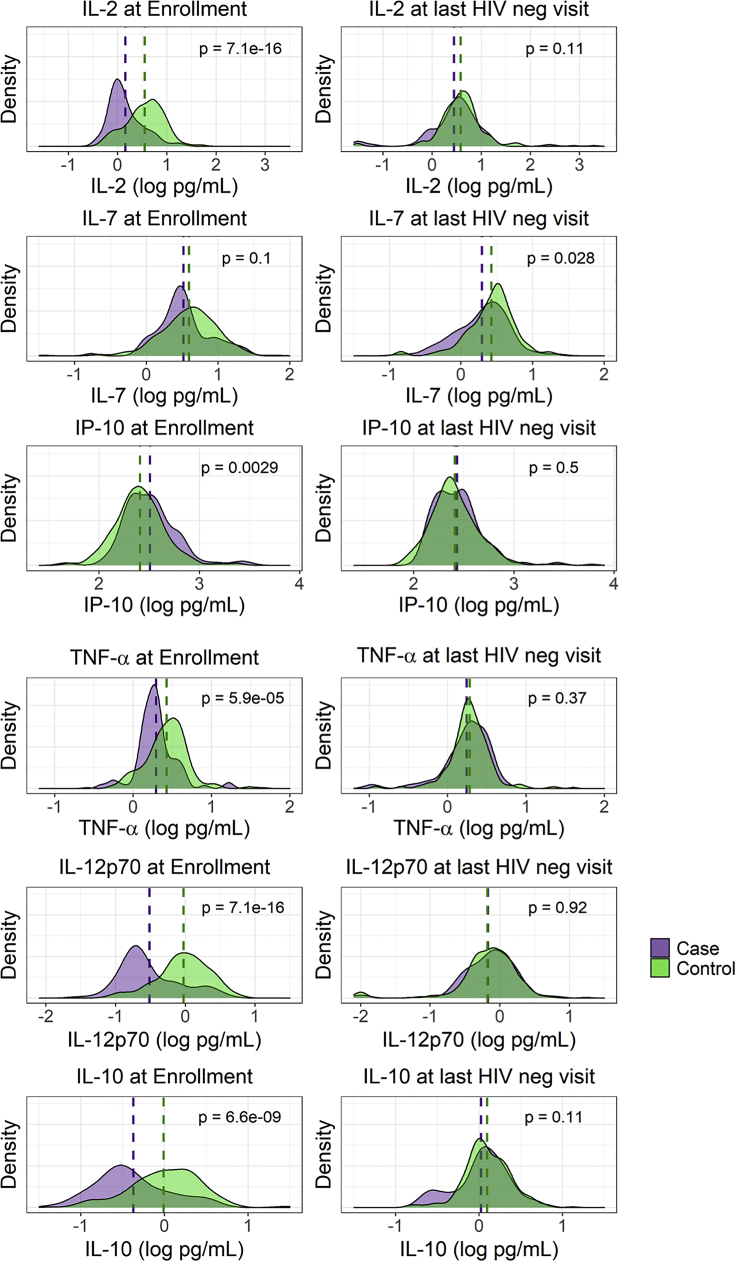
Change in Biomarkers from Baseline to last HIV negative Visit in Cases and Controls Six of 10 analyzed biomarkers are shown, as measured in cryopreserved plasma by Meso Scale Discovery multiplex chemiluminescence assay, with striking differences in the relation of case versus control distributions at study entry versus last HIV negative visit (1 month before first positive HIV RNA or Ab result or matched visit for controls). For IL-2, TNF-α, IL-12p70, IL-10 the levels of these biomarkers were significantly lower in cases than controls at study entry, but the distributions were indistinguishable between groups at the last HIV negative visit. IL-6, IFNγ, and MIP-1α were also lower in cases than controls at baseline but had a trend toward remaining lower in cases at last negative visit (not shown, p < 0.10). IP-10 was higher in cases than controls at study entry but also indistinguishable at last HIV negative visit. IL-7 had similar distributions between cases and controls at entry, but controls had higher levels of IL-7 at last HIV negative visit. TNF-β was also higher in controls than cases at last HIV-negative timepoint but was not measured in baseline samples. All data in Table 2.

**Figure 2:**
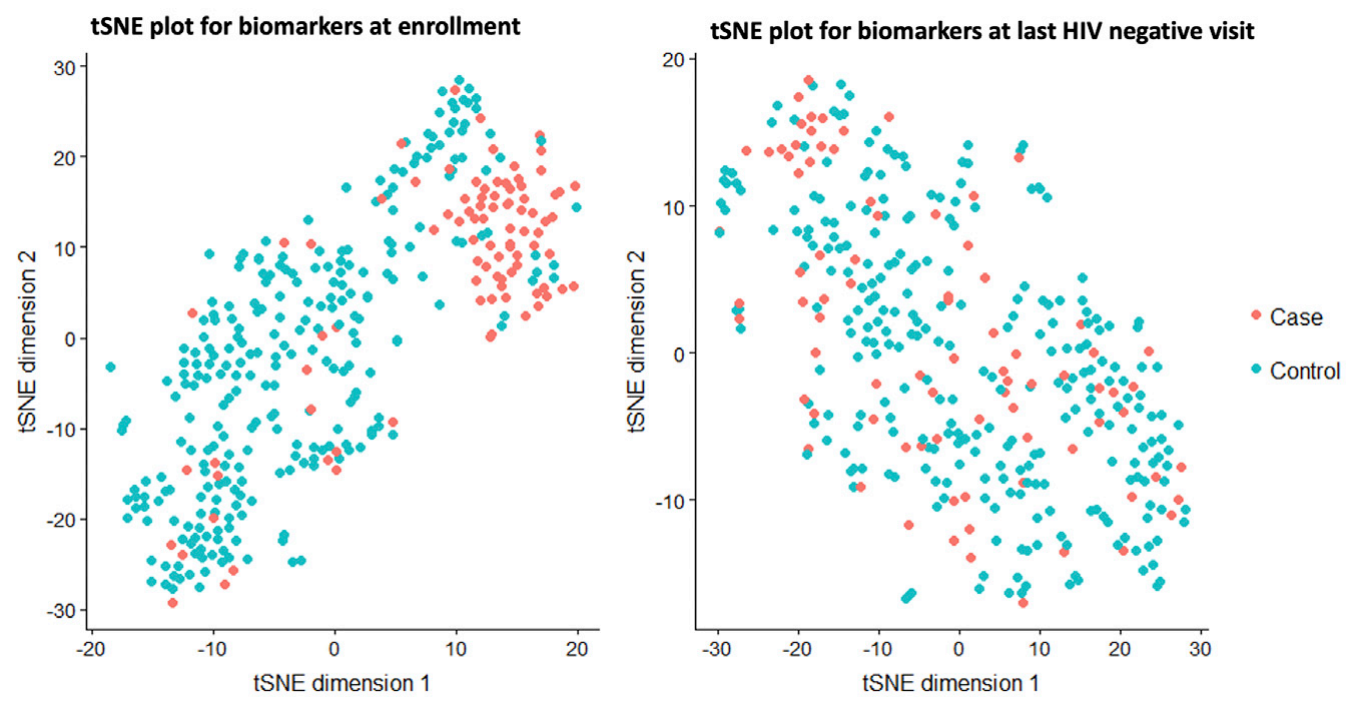
tSNE Plots show that the aggregate biomarkers for persons who did or did not later acquire HIV was highly distinct at study entry, but was indistinguishable at the last HIV negative visit T stochastic neighbor embedding (tSNE) of all 10 biomarkers collected at each visit which evaluates the total distance between individuals across the first two dimensions shows visual clustering of participants who are more similar to each other across the selected parameters. There is clear clustering of cases who would later acquire HIV at enrollment, but they are completely intermixed with controls at the last HIV negative visit.

At the last HIV negative or matched visit (X-1), IL-7 and TNF-β were higher in controls than cases; there were no other markers that differed between cases and controls, including none that were higher in cases than controls in univariate analyses (Table 2). However, at the enrollment visit, the majority of biomarkers were distinct between cases and controls, with controls having higher levels of IL-2, IL-6, IL-10, IL-12p70, IFN-γ, TNF-α, and MIP-1α. IP-10 was higher in cases than controls and TNF-β was not available at enrollment. (BH adjusted p < 0.01 for all). With the exception of TNF-α, there was a significant change in all biomarkers in cases from ENR to X-1, but in no instance did the distribution density for any biomarkers in cases exceed that of controls at X-1 (Figure 1). There was clear clustering of cases and controls into distinct groups by tSNE plot incorporating all cytokines at ENR, but no clustering at X-1 (Figure 2). Taken together, these analyses demonstrate that cases and controls had distinct profiles of soluble markers of immune activation months or years before HIV acquisition, but were largely indistinguishable in the month before HIV acquisition or matched visit in controls.

**Table 2:** Univariate estimates of cytokines at Enrollment (ENR) and the last HIV-negative visit (X-1) in cases and controls, and change in cytokines from ENR to X-1 in cases

Biomarker	Enrollment			X-1 visit			Change from ENR to X-1 In Cases	
	Cases	Controls	p value	Cases	Controls	p value		p value
IL-2 **(log**_**10**_**pg/mL)**	0.159	**0.55**	7.14 e^−16^	0.44	0.58	0.11	0.28	2.19 e−4
IL-6	0.51	**0.77**	2.81 e^−7^	0.69	0.75	0.11	0.18	1.06 e−3
IL-7	0.52	0.59	0.10	0.30	**0.42**	0.028	−0.22	5.07 e−6
IL-10	−0.37	**−0.0089**	6.61 e^−9^	0.025	0.098	0.11	0.40	1.37 e−8
IL-12p70	−0.51	**−0.028**	7.14 e^−16^	−0.17	−0.18	0.92	0.33	5.07 e−6
IP-10	**2.51**	2.41	0.0029	2.43	2.41	0.50	−0.06	0.053
IFN-γ	1.11	**1.40**	1.18 e^−7^	1.28	1.38	0.10	0.16	0.0099
TNF-α	0.29	**0.43**	5.87 e^−5^	0.24	0.28	0.37	0.05	0.22
TNF-β	–	–	–	−0.067	**0.087**	0.03	–	–
MIP-1α	1.36	**1.52**	5.87 e ^−5^	1.59	1.63	0.11	0.22	1.22 e−7

All analyses performed with univariate generalized linear models (GLM). p values are adjusted for multiple comparisons using the Benjamini-Hochberg procedure. Bolded values for ENR and X highlight the higher of the two concentrations between cases or controls when significant. All cytokine concentrations are pg/mL, log_10_ transformed.

ENR, Enrollment visit; X-1, the last HIV negative visit before the date of HIV diagnosis (X).

To evaluate possible explanations for these unexpected results, we conducted several sensitivity analyses to exclude alternate explanations. To evaluate whether time under observation or seasonality impacted biomarkers at the last HIV negative timepoint, we repeated the density plots for cases and controls, with controls stratified by how they were selected. Two of three controls for each case were selected by matching time (months) under observation, and the third control had been matched by calendar month of the X-1 visit to exclude bias from secular trends in infections or events that may have contributed to immune activation (circulating influenza or holiday-associated changes in diet or alcohol consumption, for example). There were no clear differences in the distributions of any of the biomarkers between control types or between controls and cases at last HIV negative timepoint (Figure S1).

To exclude the possibility that some of the cases might have been in the HIV eclipse phase at time of X-1 sampling,^15^ and therefore already HIV-infected but not yet viremic, we stratified cases by whether their calculated EDDI fell before or after their X-1 visit. Because the EDDI calculator incorporates all test results (HIV detected and not detected) of available methodologies, the uncertainty window around the EDDI inherently includes the possibility that a few participants who were diagnosed as seropositive were in their eclipse window if their X-1 visit was within the prior month, despite being HIV RNA-negative at that visit. If X-1 samples were in the eclipse window (EDDI before X-1, n = 20), then elevation in markers of immune activation could have been attributable to occult HIV infection rather than existing immune status at the time of exposure. The distributions of all 10 biomarkers however showed no evident differences between cases for whom EDDI was before or after X-1; all cases and controls had similar distributions, except for some markers that showed an even greater left skew (lower levels) in cases than controls (Figure S2, also as demonstrated in Table 2 and Figure 1 overall).

In addition, we explored whether regression to the mean might explain the finding that persons with extremely low or high levels of biomarkers at enrollment ended the study with more moderate levels. We hypothesized that persons who entered the study with lower levels of biomarkers, whether case or control, would be more likely to demonstrate greater changes over time. When stratified by whether their levels of each biomarker were above or below the pooled median at study entry, those with higher levels were less likely to change over time, while those with levels below the median at entry were more likely to show increases (Figure S3). For several exemplary biomarkers (MIP1-α, IL-2, TNF-α, IL-6) the change from enrollment to X-1 in cases was greater than in controls even within the below-median stratum.

**Figure 3:**
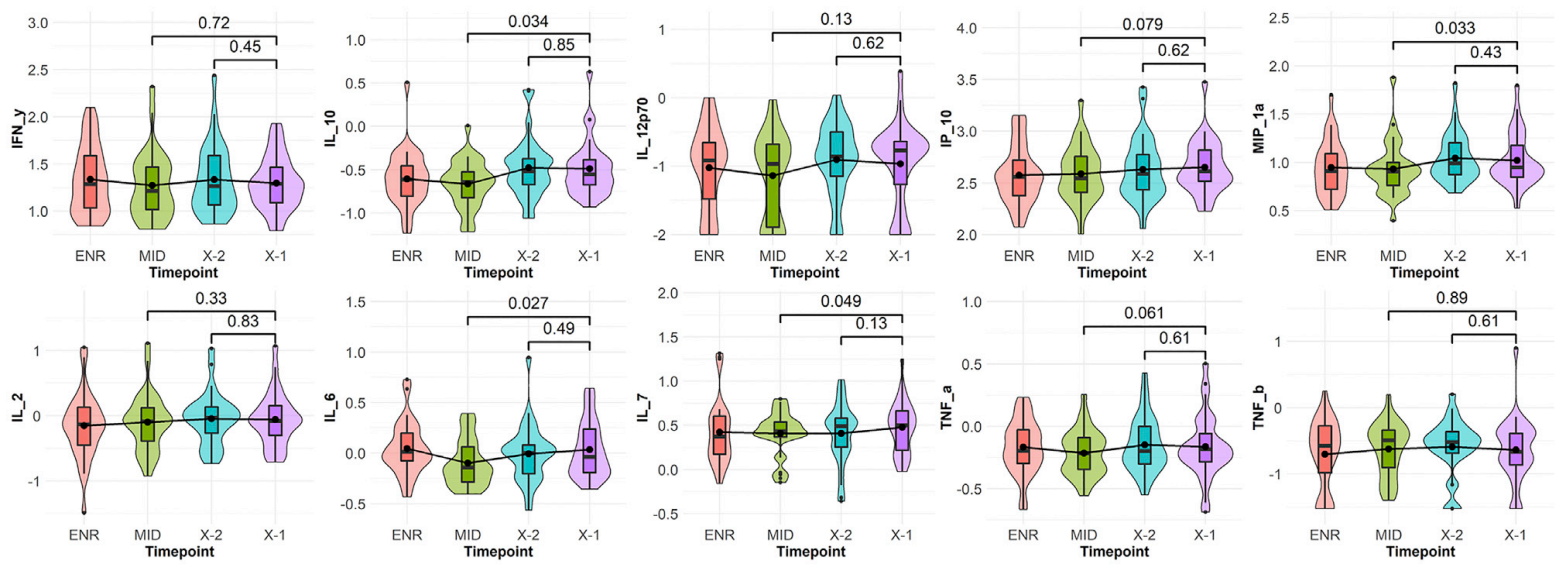
Sub-analysis timeseries in selected cases The violin plots display log_10_ transformed cytokine values in a subset of *Sabes* cases at 4 timepoints; the horizontal line represents the median and box the interquartile range, with the violin outline showing the overall density of values. We selected 30 participants who acquired HIV and repeated the baseline (ENR) and last HIV negative samples (X-1), with the addition of the penultimate HIV negative sample (X-2) as well as the sample from the month midway between ENR and X-1 (MID) on the same plate to reduce variability and to address the hypothesis that an acute change from low to high activation state could have occurred before HIV exposure, increasing susceptibility. For example, if the participant had a positive HIV RNA at Month 13, the X-1 visit was Month 12, X-2 at Month 11, and Midpoint at Month 6. Of the 30 cases, 10 were selected with X-1 occurring within the confidence window for the Estimated Date of Detectable Infection (EDDI) and 20 with X-1 outside the EDDI confidence window to verify that any inflection from X-2 to X-1 was not attributable to the X-1 sample being in an eclipse window (within 6–7 days of HIV acquisition but still HIV RNA negative; see also Figure S2). Whether overall, or stratified by time between EDDI and X-1, there was no significant change in any of the 10 markers between X-2 to X-1. However, there was an increase from MID to X-1 (median 239 days, range 63–392) in IL-10, MIP-1α, IL-6, and IL-7 with trends in IP-10 and TNF-α. Although a small sample size, this sub-analysis confirmed no effect of possible misclassification of participants in an eclipse phase and also no clear inflection point in immune activation during the HIV exposure window.

We next sought to determine whether increasing levels of biomarkers in cases occurred closer to enrollment or immediately before the HIV risk window. We therefore selected 30 cases with high variability between ENR and X-1 and repeated these two timepoints, with an additional two timepoints, all of which were run on the same MSD plate. We included the penultimate HIV negative sample (X-2; further insurance against missed eclipse phase infections and to evaluate acute biomarker change just before HIV exposure) as well as the sample from the month midway between ENR and X-1 (MID). Whether overall, or stratified by time between EDDI and X-1, there was no significant change in any of the 10 markers between X-2 to X-1. However, there was an increase from MID to X-1 in IL-10, MIP-1α, IL-6, and IL-7 with trends in IP-10 and TNF-α (at a median 239 days, range 63–392). Although a small sample size, this sub-analysis confirmed that the effect was not because of possible misclassification of participants in an eclipse phase because we did not see any participants with clear acute upticks from X-2 to X-1. We also did not demonstrate a clear inflection point in immune activation just before the true HIV exposure window, but rather a gradual increase over time, which was less marked in this non-randomly selected subset than the entire study (Figure 3).

### Risk factors for HIV exposure modeling with biomarkers

In order to understand the value of markers of immune activation to predict HIV above and beyond “traditional” demographic and behavioral factors, we then built these biomarkers into LASSO machine learning algorithms.^16^ In the first model, all covariates in Table 1 were jointly included (age, having obtained post-secondary education/training, monthly income above national minimum wage, gender identity, sexual orientation, performing sex work/transactional sex, sexual positioning, report of any condomless anal intercourse (CLAI) at study entry (yes or no), number of reported CLAI acts with non-main partners during the last 30 days before the X (HIV diagnosis, or matched) visit, participant report of any alcohol consumption (yes or no), and Alcohol Use Disorder Identification Test (AUDIT) category). This model selected younger age (OR 0.92 for each additional year above age 18, 95% CI 0.87–0.96), having a post-secondary education (OR 3.94, 95% CI 1.71, 9.07), being a versatile or receptive partner (OR 4.18 with 95% CI 1.37–12.75, and OR 4.41 and 95% CI 1.48–13.15, respectively), any CLAI (OR 5.02, 95% CI 1.64–15.34), and each additional reported CLAI act in last month (OR 1.29, 95% CI 1.12–1.49)) as increasing risk for HIV acquisition with significant coefficients. Offering transactional sex was associated with protection from HIV risk (OR 0.20, 95% CI 0.0.08, 0.49). The alcohol exposure measures, monthly income, or transfeminine identity did not predict HIV acquisition, although most covariates, except income and any alcohol consumption, were retained in the model, albeit with non-significant coefficients (Table 3A).

**Table 3:** Biomarkers and Covariates predictive of HIV acquisition using LASSO

**A. Model inclusive of all demographic risk factors**			

Variable	OR (95% CI)	p value	
Age (per year increase)	0.92 (0.87, 0.096	0.001	
Post-Secondary Education (yes)	3.94 (1.71, 9.07)	0.001	
Transgender (yes)	0.66 (0.24, 1.83)	–	
Sex Role- Versatile^a^	4.18 (1.37, 12.75)	0.012	
Sex Role- Receptive^a^	4.41 (1.48, 13.15)	0.008	
CLAI (yes at study entry)	5.02 (1.64, 15.34)	0.005	
CLAI acts in last month (non-main partner)	1.29 (1.29, 1.49)	4.53e-4	
Sex Work (yes)	0.20 (0.078, 0.49)	4.60e-4	
AUDIT 20+	1.76 (0.55, 5.63)	–	
AUDIT 8–19	1.15 (0.53, 2.49)	–	
Alcohol (Any use = Yes)	1.78 (0.86, 3.71)	–	

**B. Model with all cytokines, without demographic risks**			

ΔTNFα	1.29 (0.27, 6.21)	–	
ΔMIP-1α	0.64 (0.10, 4.15)	–	
Δ IP-10	0.12 (0.03, 0.46)	0.0020	
Δ IL-12p70	7.75 (3.33, 18.07)	2.11e-6	

**C. Model with demographic risk factors adjusted for cytokine change between ENR and X-1**			

			Percent Change from Unadjusted
Δ IP-10	0.11 (0.019, 0.67)	0.016	N/A
Δ IL-7	0.52 (0.15, 1.77)	–	N/A
Δ IL-12p70	3.56 (1.05, 12.15)	0.042	N/A
Δ IL-10	3.38 (0.81, 14.1)	0.095	N/A
Δ IL-2	1.56 (0.56, 4.32)	–	N/A
Age (per year increase)	0.92 (0.85, 0.98)	0.015	1.1%
Post-Secondary Education	6.84 (2.10, 22.33)	0.001	40.4%
Role- Versatile^a^	3.08 (0.84, 11.33)	0.090	−21.2%
Role- Receptive^a^	4.76 (1.29, 17.62)	0.019	5.3%
CLAI (yes at study entry)	3.86 (1.05, 14.23)	0.043	−16.3%
CLAI acts in last month (non-main partner)	1.35 (1.10, 1.66)	0.005	16.7%
Sex Work (yes)	0.18 (0.05, 0.58)	0.004	5.7%
AUDIT 20+	1.90 (0.44, 8.22)	–	13.4%
AUDIT 8–19	1.25 (0.47, 3.32)	–	63.2%

**A** The first model included all demographic risk factors presented in Table 1, with p values > 0.20 not presented; variables shown were selected if retained in the LASSO logistic regression model, even if the individual coefficient was not statistically significant. **B**. Model with delta of all 9 biomarkers with ENR and X-1 values available (IL-2, IL-6, IL-7, IL-10, IL-12p70, IP-10, IFN-γ, TNF-α, and MIP-1α) were included in both the unadjusted and adjusted models. Delta in biomarker signifies the change in concentration, in log_10_ pg/mL, from enrollment (ENR) to last HIV negative visit (X-1). Odds ratios (OR) represent the comparative risk of acquiring HIV per 1 log change in each biomarker; positive ORs indicate increased risk of HIV per incremental log increase in the biomarker. **C**. For the adjusted model, all cytokines and demographic covariates were included, with the ORs for the LASSO-selected covariates presented. In some cases, LASSO retained different covariates in the adjusted model than in 3A or 3B. Percent change from unadjusted compares the coefficient for that covariate in 3A vs when cytokines were included. The relationship between the demographic risk factor and HIV acquisition was considered to be modified by cytokines jointly if the change was greater than +/−15%.

All demographic covariates as reported by computer-assisted survey instrument (CASI) at study entry (all covariates presented in Table 1).

CLAI, condomless anal intercourse; AUD, Alcohol Use Disorders Identification Test with score range of 0–40, with referent range <8 signifying no or low-risk drinking, 8–19 hazardous drinking, and 20 + dependent drinking.

a Reference category: Insertive sex role (versus versatile or receptive only).

Because the changes in cytokines from ENR to X-1 were more predictive of HIV risk than either baseline or X-1 cytokines alone, we then built a LASSO model including all 9 markers for which we had measured changes over time (TNF-β not available at baseline, Table 3B). This model selected TNF-α, IL-12p70, MIP-1α, and IP-10 as jointly best descriptive of HIV risk. Positive change in IP-10 was strongly associated with decreased risk of HIV (OR 0.12 per log_10_ pg/mL increase, p = 0.0020). Positive change of IL-12p70 was associated with strong increased risk of HIV (OR 7.75, p = 2.11e-6). Although change in TNF-α and MIP-1α were important in creating a best-fit model, the individual biomarkers were not predictive of HIV acquisition in the joint model.

Next, we built a combined LASSO model including cytokines and demographic risks (Table 3C). With demographic risks included, the model selected change in IP-10, IL-7, IL-12p70, IL-2 and IL-10 as jointly predictive of HIV risk. Other markers, including two previously selected markers, TNF-α nor MIP-1α, were not selected, but IL-2, which had not previously been selected, was identified as important. Increase in IP-10 continued to remain associated with lower risk of HIV, and increase in IL-12p70 was associated with increased odds of HIV acquisition; the estimate for IL-10 was not statistically significant though tended toward a positive correlation with HIV risk (corrected p = 0.095). Adjusted for the included 5 biomarkers, older age and transactional sex work remained associated with lower risk of HIV, whereas post-secondary education, being a receptive partner, CLAI with any partner, and each additional reported CLAI act were associated with higher risk of HIV. Compared to the model without cytokines, the risk associated with post-secondary education was 40% higher. Being a versatile partner was no longer strongly associated with HIV risk, as compared to the unadjusted model, and there was effect modification (−21% change from cytokine unadjusted model). Each additional reported CLAI act now was associated with a 35% increased odds of HIV acquisition, an effect modified by inclusion of cytokines in the model (17% increase).

As a further exploration of the association between behavioral predictors, biomarkers, and HIV risk, we also evaluated change in behavioral reports between the ENR and X-1 visits. In the full LASSO model, only persons who reported engagement in sex work at baseline, but not at the X-1 visit, remained at lowest risk of HIV; persons reporting sex work near the last HIV negative visit, whether reporting sex work at baseline or not, did not have appreciably different risks. Both analyses of behavior changes had reduced power because of splitting participants into factorial groups. In the combined model, both baseline report of CLAI and quantitative number of acts were jointly selected.

## Discussion

In this carefully constructed study of MSM and transwomen considered at elevated risk of HIV in Lima, Peru, we were able to analyze plasma biomarkers of immune activation in samples collected just before the time of HIV acquisition and in matched samples from persons who did not acquire HIV, as well as from baseline samples from both groups. In most cases, samples were from within 1–3 weeks of estimated date of detectable HIV infection, and similarly within 4 weeks of HIV diagnosis. In contrast to several prior studies exploring biomarkers of HIV acquisition risk, we found no clear immune profile that distinguished cases when sampled extremely close to HIV acquisition from controls at matched timepoints. Rather, we found that most biomarkers sampled on average one year before HIV acquisition were lower in persons who later acquired HIV. For many individual biomarkers, and when the analytes were considered jointly, the most predictive factor of HIV seroconversion was the increase in the marker from baseline to the last HIV negative sample, rather than the absolute value taken at either time point. These findings were robust in both univariate analysis and joint biomarker analysis with tSNE and LASSO. We conducted several sensitivity analyses to verify that there was no confounding by accidental inclusion of samples from cases that were already in the eclipse phase of HIV infection or by other factors such as seasonality or duration of time under observation. We also did not detect an acute rise in any biomarker between the last negative sample or the penultimate samples taken one month apart in a selected subset, nor did we find overall that biomarker levels in cases exceeded those of controls. Several biomarkers that were noted to be different between cases and controls at study entry, such as IL-6, TNF-α, or IFN-γ, were not different at the last HIV-negative timepoint, and also were not selected in final LASSO models.

Although our analysis also replicated many of the demographic and behavioral risk factors that have been shown to predict HIV acquisition in diverse cohorts of MSM and transgender women (TW),^17^^,^^18^^,^^19^ we were able to demonstrate through a machine learning approach that pre-exposure changes in two biomarkers (IP-10 and IL-12p70) were independent predictors of HIV acquisition, in addition to sociobehavioral factors; IL-2, IL-7, and IL-10 contributed information to the final model, but were not independently associated. In this combined model, younger age and higher-risk sexual positioning, including report of any condomless sex and number of condomless sex acts, were associated with HIV risk as expected. Higher education level was counter-intuitively associated with increased HIV risk, perhaps because of greater socioeconomic mobility, including ability to pay for sex, attend venues like sex-on-premises night clubs, or having autonomy over living space to bring partners home, in this era before pre-exposure prophylaxis (PrEP).^20^ Gender identity was not associated with HIV risk in this analysis, which adjusted for sexual behavior and high-risk alcohol use. Although sex work and education status remained independent predictors in the model despite inclusion of reported CLAI frequency, other factors, such as partner concurrency and differential use of condoms by partnership types (e.g., main or casual partners, clients), have been previously demonstrated in this population, and may have contributed to the observations.^21^^,^^22^ These sociobehavioral risk factors were also consistent with a prior analysis done using all 2,109 persons followed longitudinally in the monthly screening cohort of *Sabes*, which also showed associations between hazardous drinking, use of amyl nitrate “poppers”, marijuana use, and increase in casual or paying partners;^23^ these clustered factors could potentially account for risk differences that are modified by immunologic parameters in the current work. Other unmeasured confounders associated with measured exposures and the outcomes could have accounted for non-intuitive relationships with HIV acquisition, including exposures that were modified by the inclusion of cytokines to our model.

Among the measured biomarkers, associations with only two biomarkers remained significant in the final models: an increase in IP-10 was associated with lower HIV risk, whereas an increase in IL-12p70 was associated with higher risk. Although greater increase in IP-10 over time was associated with lower HIV risk, it was the only biomarker that was higher in cases than controls at baseline, did not increase in most cases overall, and was indistinguishable from controls at the last HIV negative visit. IP-10, or interferon-γ inducible protein (also known as CXCL10), is not only a more stable and easily measured marker for IFN-γ activity, but has also been strongly correlated with acute disease activity in *Mycobacterium tuberculosis*, hepatitis C virus, herpes simplex virus, and other pathogenic infections that elicit a strong Th1 response.^24^^,^^25^^,^^26^^,^^27^ IP-10 is induced in response to HIV,^28^ has been shown to promote HIV replication,^29^ and is also associated with increased T regulatory activity and immune non-response in treated HIV.^30^ Related observations are the possible protective role of T-regulatory cells among highly-exposed persons who remain seronegative,^31^ and that a lower level of mucosal IP-10 is also associated with HIV resistance in that population.^32^ One possible explanation is that IP-10 assists in recruiting CD8^+^ T cells to the mucosa to promote immunosurveillance^33^ and simultaneously downregulates proliferation of HIV-susceptible CD4^+^ T cells.^34^ Because prior studies evaluating plasma biomarkers and HIV risk sampled cross-sectionally at various times prior to infection, it is likely that the heterogeneity in sample acquisition time contributed to somewhat conflicting findings between studies, including the variable directionality or lack of effect of IP-10 on HIV risk.^4^^,^^6^

Conversely, although IL-12 also ultimately results in increased IFN-γ induction, it is primarily produced by dendritic cells and results in activation of CD4^+^ T cells and NK cells.^35^ Therefore, increase in plasma IL-12 likely represents a temporal increase in a pool of HIV-susceptible cells. Both the Partners in Prevention and CAPRISA-004 studies, neither of which included samples very close to EDDI, reported that IL-12 was associated with HIV acquisition. However, in a recent analysis of serodiscordant heterosexual couples from Rwanda and Zambia (RZHRG study), absolute levels of IL-12 were not associated with HIV acquisition in samples taken on average 45 days before estimated date of infection; neither IP-10 nor IFN-γ were assessed in that analysis.^36^ We found no difference in IL-12 levels proximal to EDDI, but found that increases over time were strongly associated with higher risk. We also confirmed the RZHRG finding that elevated IL-7 just before EDDI was a risk factor, but the individual contribution of the change in IL-7 level on HIV acquisition did not remain significant in LASSO. Although several traditional risk factors contributed to the final models, and inclusion of IL-2, IL-7, IL-10, IP-10 and IL-12 added additional predictive value, the effects of many of the sociobehavioral risk factors were not significantly modified by the biomarkers. Notably, the magnitude of effect of CLAI and sexual positioning was altered when biomarkers were included in the model. Therefore, there are likely mechanistic pathways involving local and systemic inflammation associated with some exposures, whereas the effect of others would not be expected to be mediated by biomarkers, unless through other exposures also associated with socioeconomic status. In a sub-study among the persons in this same cohort who acquired HIV and entered into the randomized treatment study, we found moderately strong associations (1.3– to 1.7-fold differences) between levels of the alcohol metabolite phosphatidylethanol (PEth) and IFN-γ, TNF-α, and IL-12. Persons with HIV who smoked also had 1.4– to1.8-fold higher levels of TNF-α, MIP-1α and IL-12, compared to non-smokers.^14^

Future work is needed to evaluate preventable causes of intrinsic and extrinsic immunologic risk for HIV, especially factors which trigger interferon pathways and T cell activation. Thus far, HSV-2 serostatus has shown strong associations with HIV risk, even among those who have asymptomatic infection.^37^^,^^38^ However, suppression of HSV-2 with acyclovir was not shown to decrease HIV risk.^5^ Schistosomiasis and filariasis have also been identified as risk factors for both urogenital inflammation and HIV acquisition in co-endemic regions, although more recent studies have failed to show an association between infection with these parasites and the systemic immune activation that predicted HIV risk in the RZHRG study.^36^^,^^39^^,^^40^ Neither of these pathogens are present in the geographic region in which our study took place, although persons in this cohort could have had differential risk of exposure to malaria and arbovirus infections by socioeconomic status. Separately, we performed the first study, to our knowledge, evaluating whether *M. tuberculosis* infection increases HIV risk, in a subset of Step vaccine study participants. In that study, we found no association between sub-clinical *Mtb* infection and HIV acquisition. However, we found associations with increased transcription of certain gene profiles, including interferon sensitive genes, and HIV risk.^41^ Preliminary work in our cohort showed that the seroprevalence of cytomegalovirus (CMV), HSV-1, and HSV-2 was very high, as expected, but there was no increased risk of HIV acquisition associated with baseline serostatus of these viruses. Our group is now working to identify whether prevalent chronic or interval acute viral infections could have contributed to immune activation during the risk window with an innovative high-throughput virologic technique and longitudinal sampling. Our finding that the change in concentrations of soluble molecules was associated with HIV acquisition risk is intriguing. However, we do not yet fully understand the underlying component causes. Because it was the change in these concentrations over time that was most strongly associated with HIV risk, it is less likely that persistent immune states contributed to our findings. Instead, changes in sexual behavior and substance use, interval acquisition of infections, or other state changes contributed to the findings. Regardless, identification of infections that can be prevented or treated is an important step in reducing HIV risk, in addition to the urgent need for availability and accessibility of PrEP. Attention to high-risk systemic immune profiles also remains an important consideration for the development of HIV or other vaccines, to prevent the possibility that certain vaccines could elevate HIV risk, as was seen in the Step study.^1^

### Conclusions

Dynamic changes in immune states are associated with HIV acquisition, and biomarker and demographic and behavioral data add complementary information to HIV risk. Identification of and interventions for conditions that contribute to high-risk systemic immune profiles remains an important consideration to augment HIV prevention strategies, and is an important consideration for vaccine development.

### Limitations of the study

Limitations of our study include that we were unable to assess cellular markers because of the high burden and complexity entailed in collecting monthly PBMCs in more than 2,000 participants over 20,200 longitudinal visits. We therefore used a carefully selected panel of 10 soluble markers that we measured in plasma. Because of the nature of samples and data from the parent cohort, we also did not have detailed data on anogenital infections or mucosal immune samples over time, preventing, for example, assessment of the impact of prior or incident syphilis infection, a disease which is known to cause systemic and gastrointestinal inflammation. Although we acknowledge that lack of mucosal samples limits our ability to correlate local and systemic immune responses, this study was designed explicitly to address the potential impact of systemic immune markers feasibly sampled in peripheral blood. Apart from frequency of condomless anal intercourse, we did not have other details on sexual encounters to be able to correlate our findings with anogenital infections and genital inflammation, which have been strongly associated with HIV risk, predominately in cisgender women.^42^^,^^43^ Work on associating viral infections with at-risk systemic immune profiles is ongoing by our group. Because this project included over 840 samples, and baseline samples were received first, sample analysis was completed in two batches. We however mitigated the risk of batch effect by carefully balancing cases and controls on plates, performing the described sensitivity analysis, and rerunning a subset of samples. The strengths of this study include longitudinal sampling of plasma as well as detailed data on sexual behavior and substance use.

## STAR★Methods

### Key resources table

**Table:** 

REAGENT or RESOURCE	SOURCE	IDENTIFIER
**Biological samples**		

Human Plasma	Sabes Clinical Study^11^^,^^46^	NCT01815580(author’s own)

**Critical commercial assays**		

MSD Custom U-PLEX Multiplex Biomarker Assay (IL-2, IL-6, IL-7, IL-10, IL-12p70, IP-10, IFN-γ, TNF-α, TNF-β, and MIP-1α)	Meso Scale Diagnostics^44^	K15067L-2

**Deposited data**		

Dasgupta, Sayan (2022), “Immune activation and other risk factors for HIV acquisition”	Mendeley Data, V1; This paper	https://doi.org/10.17632/8k97yxxghm.1

**Software and algorithms**		

R. Studio	Open Source, Boston, MA	RStudio Version 1.3.1093

### Resource availability

#### Lead contact

Further information and requests for resources and should be directed to and will be fulfilled by the lead contact, Dr. Rachel Bender Ignacio (rbi13@uw.edu).

#### Materials availability

This study did not generate unique reagents or other materials.

### Experimental model and participant details

#### Study participant population

Between 2013 and 2017, the parent *Sabes* study evaluated an HIV treatment-as-prevention intervention among persons assigned male at birth who have sex with men (MSM and transgender women, TW), the populations most affected by HIV in Lima, Peru. The three-step screen, re-screen, and treat study design has been published. In brief, of 2,685 MSM and TW who were HIV-uninfected but at high-risk for HIV, 2,109 entered a longitudinal cohort and were tested monthly by point-of-care third-generation HIV immunoassay and for HIV-1 RNA by pooled NAAT test if negative.^11^ Only persons assigned male at birth and not taking feminizing hormones were included in the parent study because analysis included measurement of HIV RNA in semen to evaluate the treatment as prevention intervention. Pre-exposure Prophylaxis for HIV (PrEP) was not available outside of clinical trials at this time in Peru, no Sabes participants received PrEP or post-exposure antibiotic prophylaxis for bacterial sexually transmitted infections (STIs) during this study, but were offered treatment for diagnosed STIs. We created a nested case-control study by identifying as cases, 90 participants who were diagnosed with incident HIV, either while seronegative, or within 3 months of last negative test. Cases were matched 3:1 to controls. Two controls were matched by time under observation, meaning that they were followed for at least the same number of months and had available samples at matched study visits. The third control was matched by calendar month to account for seasonal variation in exposures. Defining the date of HIV diagnosis as X, we selected cryopreserved plasma from the visit one month prior to HIV diagnosis or matched visit (X-1) as well as the baseline enrollment visit (ENR). Due to matching on time and desire to evaluate whether biomarkers mediated demographic and behavioral risks, we did not perform propensity score-based matching.

All participants completed an Alcohol Use Disorder (AUDIT) questionnaire at enrollment and answered detailed demographic and sexual behavior questionnaires. At each subsequent visit HIV-uninfected participants answered similar questions about ongoing exposures via a Computer Assisted Survey Instrument (CASI). We used the CEPHIA algorithm to calculate the Estimated Date of Detectable Infection (EDDI; most probable first date of viremic HIV infection), which is a published calculator that gives an uncertainty window ranging from Earliest Possible to Latest Possible Date of Detectable Infection and provides more precision than Fiebig staging or other methods of evaluating recency of HIV acquisition.^13^

This study was approved by the University of Washington Human Subjects Division; the parent study was approved by the Fred Hutchinson Cancer Research Center IRB, the Comité Institucional de Bioética de Asociación Civil Impacta Salud y Educación and the Peruvian Instituto Nacional de Salud. All participants in the parent study gave consent for future use of stored samples.

### Method details

#### Laboratory methods and rationale

We used a custom Meso Scale Discovery (MSD) U-PLEX chemiluminescent immunoassay panel (Meso Scale Diagnostics, Rockville, MD) to test 10 soluble biomarkers selected to encompass innate and T-cell responses to viral infections. Prior studies with contrasting results (Partners PrEP and CAPRISA-004) used Luminex, and it is possible that the platform for cytokine analysis may have contributed to the between study variability.^4^^,^^6^ Prior comparisons of Luminex kits demonstrated that while relative patterns in cytokines were similar among kits, there were highly significant differences among lots or laboratories working with the same samples; results were not reproducible enough to compare repeated determinations over time,^44^ and Luminx can be hampered by inhibitors that decrease sensitivity.^45^). We (Hladik at al, unpublished data) also found that MSD performes at a broader analytic range of up to 3 logs and improves sensitivity compared to Luminex.

In our 10-plex custom pane, we tested the following biomarkers: IL-2, IL-6, IL-7, IL-10, IL-12p70, IP-10, IFN-γ, TNF-α, TNF-β, and MIP-1α in cryopreserved plasma from ENR and the X-1 visit, as previously published.^46^ Rationale for selected markers included: signlas with IL-2, IL-7, IL-12, TNF-α, IL-10, and IP-10 in either CAPRISA-004 or Partners in Prevention or both. IFN-γ was selected to pair with IP-10 due to findings of risk in the Step vaccine study. To round out the assay, we selected 3 further markers implicated in pro-inflammatory responses, specifically with respect to monocyte/macrophage, and T-cell activity (MIP-1α, IL-6, TNF-β).

Samples were run in duplicate and values checked for variance and averaged for use, assuming quality checks were passed. Samples were run on plates with cases and controls evenly admixed for both timepoints. The TNF-β analyte was not available on the ENR samples, except the longitudinal subanalysis of 30 participants, for which all four timepoints from a single participant were run on the sample plate. All samples had gone through a single additional freeze-thaw for aliquoting prior to analysis. Protein concentrations were determined using MSD Discovery Workbench (version 4.0) analysis software. The light intensities from the samples were interpolated using a four-parameter logistic fit to a standard curve of electrochemiluminescence generated from known concentrations. The lower limit of detection for each marker can be found on the manufacturer’s website.

### Quantification and statistical methods

Given that this is a Case-Control study, we used conditional logisitic regression to evaluate associations between HIV acquisition and each biomarker considered independently using the values from ENR, X-1, and the change in each marker between ENR and X-1. We also evaluated the marginal association between each baseline covariate (age, education, gender identity, sexual orientation, participation in sex work, AUDIT score, report of condomless anal intercourse (CLAI), and sexual role (insertive or receptive partner exclusively, or versatile). We focused on sexual behavior and substance use covariates previously associated with HIV acquisition in an analysis of the full cohort and a similar study in Peru by our group^21^^,^^22^; there was insufficient prevalence of substances other than alcohol (<10% marijuana use and <1% cocaine, opioids, or methamphetamine) to include those as predictors. Sex and race/ethnicity were not used as covariates; all participants were asigned male at birth and all participants self-identified as “mestizo” or mixed-race/Latinx. For CLAI, we used both baseline report of currently practing any CLAI (binary; yes or no report at study entry), and a variable that compiled counts of individual CLAI acts across different types of partnerships, as reported by CASI, asking participants to recall the last 30 days prior to the study visit. For this variable, we selected the participant’s total number of CLAI acts with non-main/primary partners from the “X” visit, or the visit at which HIV was diagnosed for cases or matched control timepoint; data had been recorded prior to receiving HIV testing results to minimize reporting bias in recall of the 30 days of the presumptive risk window since the X-1 visit. Based on prior work in our group and other cohorts, it has been shown that there is differential participation in condomless acts between main or primary partners and other types of partnerships (casual, anonymous, clients etc), and that there is differential HIV risk associated with CLAI in different partnerships.^21^^,^^22^^,^^47^^,^^48^ Therefore, in this study, we used the time-updated count of CLAI acts with all non-main partnership as the exposure of interest. We also explored use of all reported CLAI acts, but found this variable to be less predictive, as expected, and also created distortions in models.

Acknowleding the high collinearity and not always clear relationships between soluble biomarkers in different immunologic pathways, we visualized the joint effects of all biomarkers together using t-distributed Stochastic Neighbor Embedding (tSNE) plots^49^ to display the joint contrasts between cases an controls. To reduce dimensionality of the data and select cytokines jointly predictive of the outcome, we used LASSO (Least Absolute Shrinkage and Selection Operator) with logistic regression to model the outcome.^16^^,^^50^ LASSO was chosen both to reduce the multi-colinearity of the biomarkers and traditional risk factors and for variable selection. LASSO uses L1 regularization on the regression parameters to achieve variable selection and enhanced prediction accuracy and interpretability of the resulting statistical model. The amount of penalization/regularization (controlled by the parameter λ) was selected through a 10-fold cross-validation. We created two nested datasets, one with demographic and clinical covariates, and the other with demographic and clinical covariates plus all cytokines, and ran LASSO to select a final model for each. Effect modification was determined by assessing a 15% or greater change in the covariate effect size when cytokines were included in the model. For the sub-analysis, we created violin plots and used paired t-tests between timepoints for cases only.

To further analyze longitudinal changes over time, we selected 30 cases and 10 controls and selected plasma from the ENR, X-1 visit, X-2 visit (2 months prior to HIV diagnosis or matched timepoint), as well as the visit that was midway between ENR and X (diagnosis date). We selected the 30 cases with the greatest ranked sum of changes in biomarkers between ENR and X-1. Of these cases, 20 definitively acquired HIV following the X-1 visit per the CEPHIA calculator, while the last 10 were designated by the calculator as possibly in the eclipse phase at the X-1 visit (by definition, all cases and controls were negative for HIV RNA at the X-1 visit, but the calculated EDDI may have fallen prior to X-1). For this sub-analysis, we repeated all timepoints of each case and control on the same plate.

All analyses were performed in R studio (RStudio Version 1.3.1093, Open Source, Boston, MA).

## Data Availability

• De-idenditifed datasets and code have been deposited in Mendeley and are publicly available as of the date of this publication. Accession numbers are listed in the key resources table. • Any additional information required to reanalyze the data reported in this paper is available from the lead contact upon request.

## Supplemental information

Document S1. Figures S1−S3
