# Supplementary material for: Dynamic immune markers predict HIV acquisition and augment associations with sociobehavioral factors for HIV exposure
Source: iScience. 2022 Nov 19;25(12):105632. doi: 10.1016/j.isci.2022.105632 (PMC9722478; doi:10.1016/j.isci.2022.105632)
Supplement: Document S1. Figures S1−S3 [file mmc1.pdf]

iScience, Volume *25*

Supplemental information

Dynamic immune markers predict HIV acquisition and augment associations with sociobehavioral factors for HIV exposure

Rachel A. Bender Ignacio, Sayan Dasgupta, Rogelio Valdez, Urvashi Pandey, Siavash Pasalar, Ricardo Alfaro, Florian Hladik, Germán Gornalusse, Javier R. Lama, and Ann Duerr

Supplemental Information

Tables and Figures

Figure S1: Biomarker distributions at the last HIV negative timepoint in Cases and Controls, stratified by control type, pertains to RESULTS section and Table2 and Figure 1

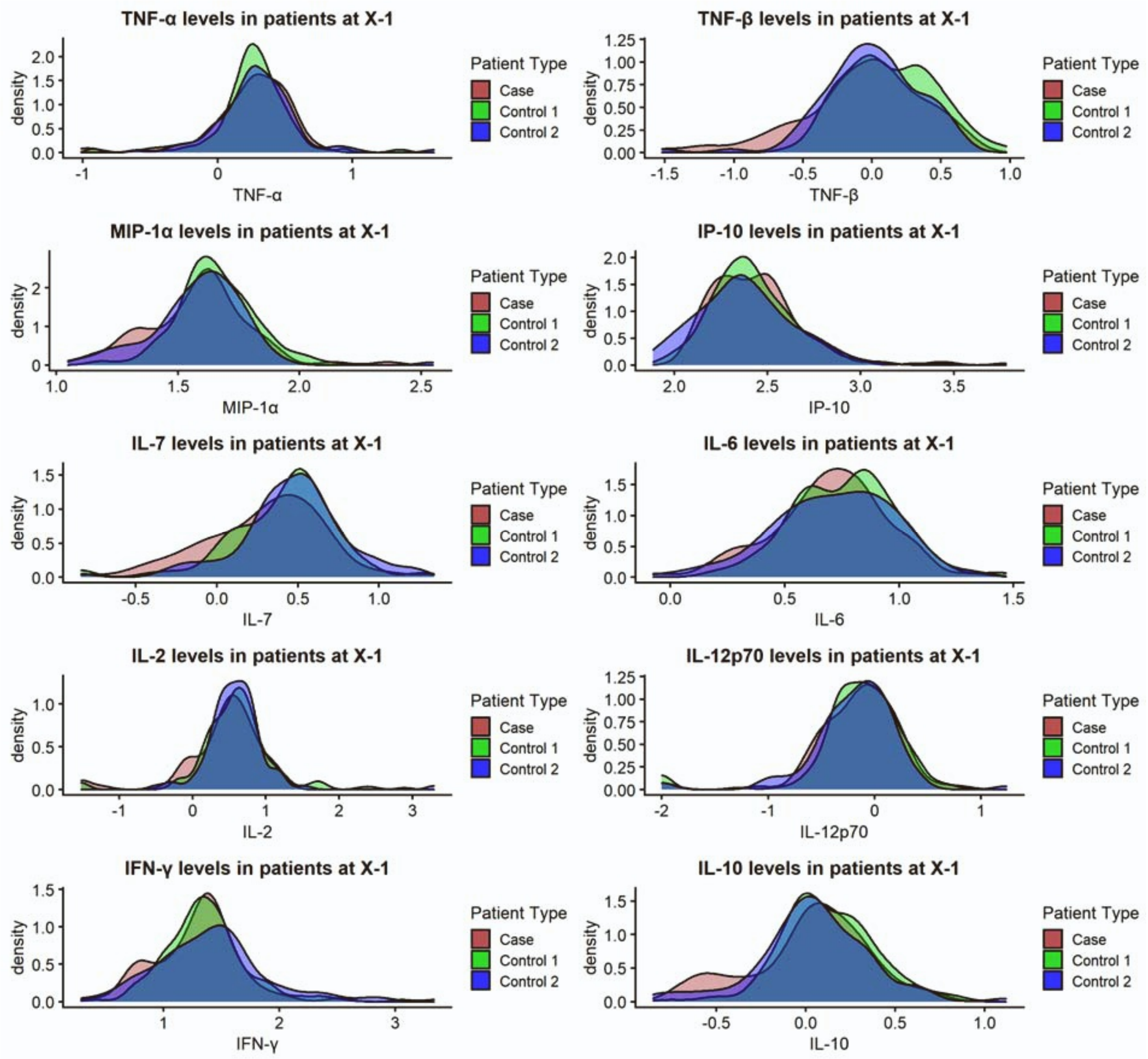

To evaluate whether time under observation or seasonality impacted biomarkers at the last HIV negative timepoint, we repeated the density plots for cases and controls, with controls stratified. Control 1 were controls selected for the same time under observation as the case (if the case was diagnosed with HIV at Month 13, the Month 12 sample was selected for both case and control). Control 2 were samples in which the X-1 visit fell within the same calendar month, to exclude bias from secular trends in infections or events that may have contributed to immune activation (circulating influenza or holiday-associated changes in diet or alcohol consumption for example). There were no clear differences between Controls 1 and 2 or cases at last HIV negative timepoint.

Figure S2: Distribution of biomarkers among Cases and Controls, with Cases stratified by timing between Estimated Date of Detectable Infection (EDDI) and sample identified as last HIV negative Visit, pertains to RESULTS section and Tables 2 and Figure 1.

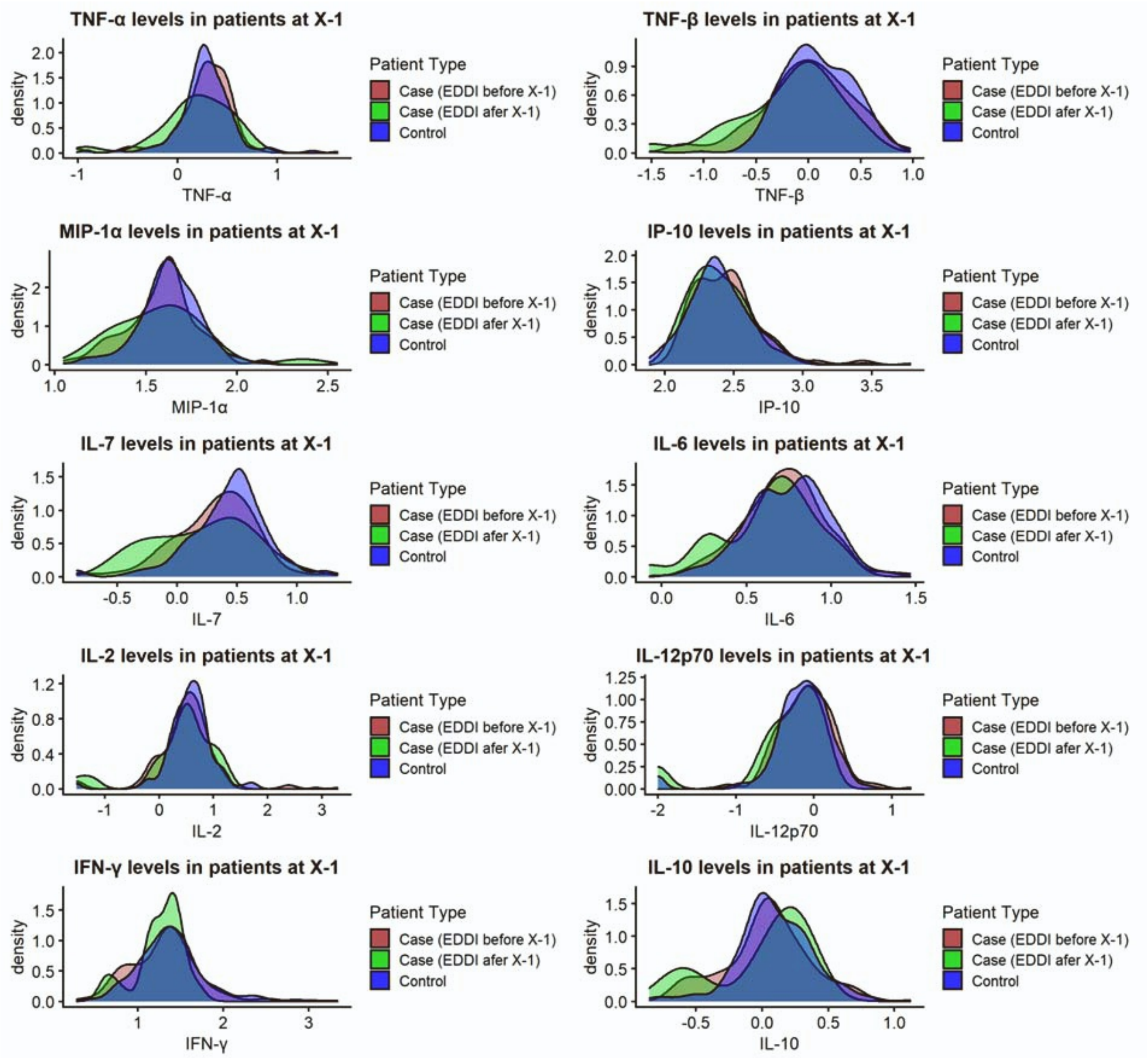

The last HIV negative sample (X-1) was selected based on retrieving the sample from one month (on average) prior to the first detection of HIV by RNA or positive antibody testing, meaning that the X-1 sample was by definition RNA and antigen negative. Because the EDDI calculator incorporates all available test results (HIV detected and not detected), unless the first HIV positive result was antibody positive or sufficiently distant from the X-1 sample, the uncertainty window around the EDDI inherently includes the possibility of being in the eclipse window (HIV infected, not yet detectable plasma RNA). If X-1 samples were in the eclipse window (EDDI before X-1, n=20), then any increase in markers of immune activation could have been attributable to occult HIV infection. However, the below distributions of all 10 biomarkers show no evident differences between cases for whom EDDI was before or after X-1 (n=70); overall cases and controls have similar distributions, except for some markers that show an even greater left skew (lower levels) in cases than controls as demonstrated in Table 2 and Figure 1.

Figure S3: Changes in Cases and Controls from Enrollment to last HIV negative visit (ENR to X-1), stratified by baseline biomarker level, Pertains to RESULTS section and Table 2 and Figure 1.

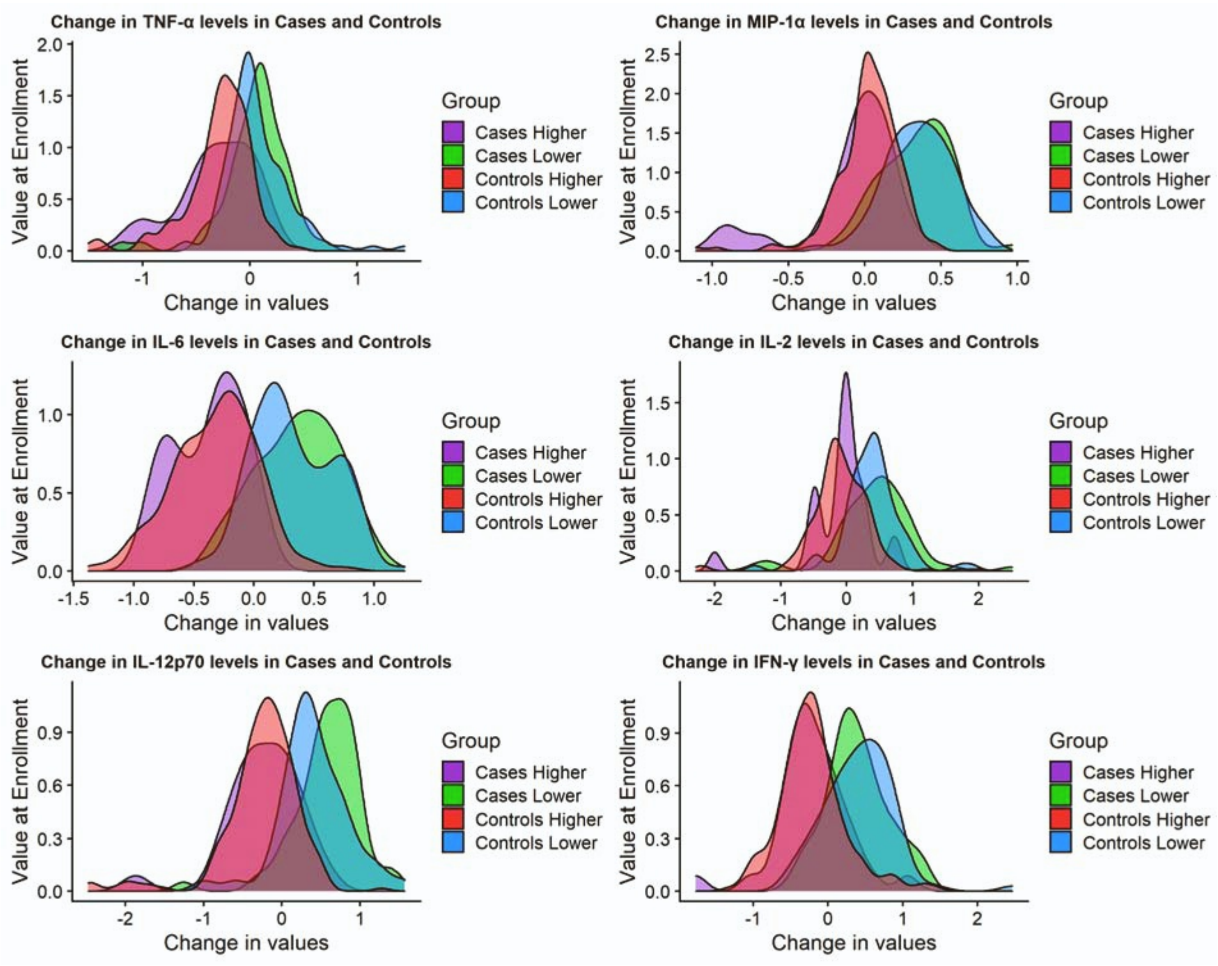

To evaluate whether persons (either cases or controls) who entered the study with lower levels of biomarkers were more likely to have a greater change over time, we stratified cases and controls by the median level for each biomarker and plotted the distribution plots separately. The six biomarkers with greatest change from ENR to X-1 are plotted below, the four others not shown. For all six biomarkers, both cases and controls with higher levels at ENR (purple and red, respectively) showed less change as a group than cases and controls with lower baseline levels (green and blue, respectively). While it is possible that this represents regression to the mean to some extent taken all together, the data reflects the idea that those with large changes in immune activation are highest risk for HIV acquisition, regardless of baseline levels. Therefore, it may not be the absolute level that is associated with risk, but rather the change that is either demonstrating a process associated with susceptibility or else a biologic process that is associated with both developing immune activation and also HIV susceptibility.
